# The Predictive Value of Preoperative Histological Risk Factors in Early Cervical Cancer

**DOI:** 10.3390/jcm14103277

**Published:** 2025-05-08

**Authors:** Jana Adams, Amelie Wingels, Constanze Amir-Kabirian, Janice Katharina Jeschke, Lara Gesemann, Büsra Eser, Caroline Lenz, Bernd Morgenstern, Fabinshy Thangarajah

**Affiliations:** 1 Department of Gynecology and Obstetrics, Medical Faculty, University Hospital of Cologne, University of Cologne, 50937 Cologne, Germany; jana.adams@uk-koeln.de (J.A.);; 2 Department of Gynecology and Obstetrics, Medical Faculty, University Hospital of Münster, University of Münster, 48149 Münster, Germany; 3 Department of Gynecology and Obstetrics, Medical Faculty, University Hospital of Essen, University of Essen Duisburg, 45147 Essen, Germany

**Keywords:** cervical cancer, histopathology, grading, prediction, accuracy, concordance

## Abstract

**Background**: Cervical cancer is a leading cause of morbidity and mortality among women globally. Currently, treatment is primarily based on tumor staging; however, discrepancies between preoperative and postoperative tumor staging remain a significant challenge and may impact treatment decisions and outcomes. This study aims to investigate the disparity between preoperative and postoperative risk factors in early-stage cervical cancer, with a particular focus on the histopathological parameters and the correlation with preclinical risk factors. **Methods**: Patients who underwent surgical treatment for an initial diagnosis of primary cervical carcinoma at the University Hospital Cologne in the Department of Gynecology and Obstetrics between 2015 and 2021 were included. A retrospective analysis was conducted to examine variations in histological parameters and their relationships with preclinical risk factors, such as age, BMI, smoking status, and HPV status, along with pretherapeutic diagnostic results. **Results**: In 85.7% of cases, preoperative grading showed concordance with postoperative grading. Postoperative upgrading was observed in 14.3% of cases with no instances of downgrading. Inconsistent findings were noted for venous invasion (3.6% of cases) and lymphovascular space invasion (6.7% of cases). No significant correlations were found between pre- and postoperative discrepancies and preclinical risk factors or pretherapeutic diagnostics. Kaplan–Meier analyses revealed no impact of discordance in grading (*p* = 0.559) or lymphatic vessel invasion (*p* = 0.752) on recurrence-free survival. **Conclusions**: The analyzed discrepancies were not influenced by preclinical risk factors or pretherapeutic interventions and showed no significant prognostic relevance for the patients’ recurrence-free survival. More robust conclusions would require further studies with larger sample sizes.

## 1. Introduction

Cervical cancer is one of the most prevalent malignancies among women worldwide, with an estimated 604,000 new cases and 342,000 deaths annually [1]. Therapy for cervical cancer is primarily determined by the tumor stage, which includes the size of the tumor (T), regional lymph node involvement (N), and the presence of distant metastases (M) [2]. Treatment options typically include surgery, radiation therapy and chemotherapy, either as standalone therapies or in combination depending on the stage of the disease at diagnosis [3]. In Germany, treatment recommendations are governed by the updated national guidelines, which emphasize a personalized approach to therapy based on tumor staging, histological characteristics and the patient’s overall health. These guidelines recommend surgical approaches in early-stage cancer and chemoradiation in advanced stages [4].

Ultimately, treatment decisions are heavily dependent on accurate preoperative diagnostics, which include imaging techniques, clinical examinations and histopathological findings. The purpose of these preoperative evaluations is to determine the scope of tumor dissemination and to guide surgical decision making. Nevertheless, discrepancies between pre- and post-treatment tumor staging can occur and remain a challenge. The accuracy of preoperative assessments is critical, as incorrect staging can lead to suboptimal therapy and a worse outcome.

In other gynecological tumor entities, such as endometrial or breast cancer, preoperative histology is only a modest predictor and tends to underestimate the extent of the disease [5,6,7,8,9]. For cervical cancer, data are rare. Agreement or discrepancy in terms of clinical over- or underestimation has mainly been investigated with regard to the pre- and postoperative tumor staging (T) [10,11,12,13]. It has been shown that an inconsistency has prognostic significance for patients. Patients with clinically underestimated tumor staging had a higher recurrence rate and worse overall survival compared to those in whom the tumor stage was either clinically correctly assessed or overestimated [10].

Preoperative core needle biopsy can accurately distinguish histological subtypes, with a high sensitivity for squamous cell carcinoma and adenocarcinoma but lower sensitivity for adenosquamous carcinoma. The prediction of lymphovascular space invasion (LVSI) has an accuracy of 62%, influenced by tumor size and inflammation, while tumor grade (G3) can be diagnosed correctly in 74% of cases, affected by the histological tumor type [14]. Preoperative underassessment can compromise the effectiveness of cervical cancer surgery, leading to deviations from recommended excision guidelines and inaccuracies in disease staging [13].

Several factors contribute to these discrepancies, including limitations in imaging technologies, pathological misinterpretations and variations in the clinical expertise of the treating team. Moreover, the subjective nature of histopathological assessments and the heterogeneity of tumor types may further complicate preoperative decision making. This gap in preoperative and postoperative staging information emphasizes the need for improved diagnostic tools and more reliable methods for tumor assessment.

This paper aims to investigate the predictive value of preoperative diagnostics of grading, lymphovascular invasion, and venous invasion. We aim to explore the discrepancies between pre- and postoperative findings, the impact of these discrepancies on therapeutic outcomes, their effect on recurrence-free and overall survival, and strategies to reduce the future risk of erroneous treatment decisions.

## 2. Materials and Methods

Patients who underwent surgical treatment for a primary diagnosis of cervical carcinoma at the Department of Gynecology and Obstetrics, University Hospital Cologne, between 2015 and 2021, were included in this study. All patients received a biopsy prior to surgical treatment. Patient identification was conducted using the ODSeasy system (astehnis^®^ medical GmbH, 2022, Aschheim, Germany). Clinical data were retrospectively collected from medical records. Statistical analyses were performed using IBM^®^ SPSS statistics (version 30.0, Armonk, NY, USA), employing Fisher’s exact test for correlation analysis and log-rank test for Kaplan–Meier curves.

## 3. Results

**Figure 1:**
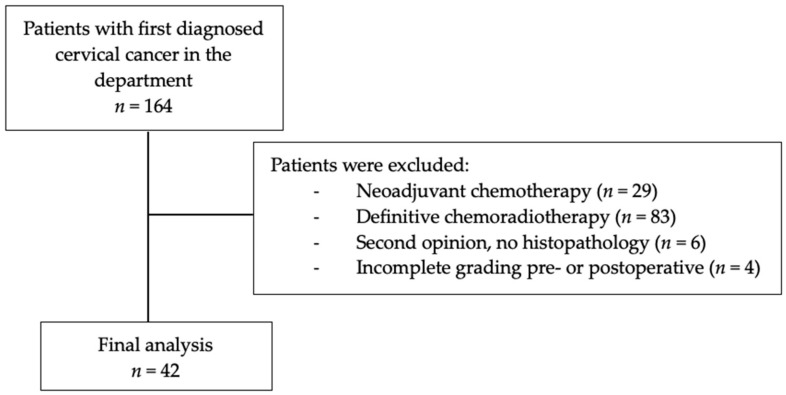
Selection of patients for final analysis.

In total, 42 patients met the inclusion and exclusion criteria and were included in the final analysis (Figure 1).

### 3.1. Demographic Data

Patients’ demographic data are presented in Table 1. The median age at first diagnosis was 45 years [36–54], and the median body mass index (BMI) was 25.1 [20.7–26.4]. A quarter of the patients (*n* = 9) were smokers, while 75% (*n* = 26) were non-smokers. An infection with HPV was detected in 96.4% of patients (*n* = 27), of whom 64.3% (*n* = 18) were infected with HPV16, 7.1% (*n* = 2) with HPV18 and 25% (*n* = 7) with other HPV types. Only one patient tested negative for HPV, and no information was available for 14 patients regarding their HPV status. In the overall cohort, 62.5% of the patients (*n* = 25) underwent a cone biopsy (conization), while 37.5% (*n* = 15) did not. No information was available for two patients.

The most common clinical FIGO stage was stage I found in 85.2% of cases (*n* = 23). For 15 patients, no clinical FIGO stage was documented.

Regarding the postoperative pathological T-stage, the vast majority of patients (95.2%, *n* = 40) were diagnosed with pT1. Similar to the clinical FIGO stage, no patient was diagnosed with a pathological T-stage higher than 2.

### 3.2. Pre- and Postoperative Histopathological Findings

Results of the pre- and postoperative grading (G) venous invasion (V) and lymphovascular space invasion (LVSI) are presented in Table 2.

To assess discrepancies, the pre- and postoperative classifications were compared and categorized as overestimation when the preoperative G, V, and LVSI stages were higher than the postoperative ones and as underestimation when they were lower. Regarding preoperative grading, 14.3% of patients (*n* = 6) were classified as G1, 59.5% (*n* = 25) as G2, and 26.2% (*n* = 11) as G3. Postoperatively, 7.1% of patients (*n* = 3) were classified as G1, 59.5% (*n* = 25) as G2, and 33.3% (*n* = 14) as G3.

Using the binary grading system, 73.8% of patients (*n* = 31) were categorized as G1 + G2 and 26.2% (*n* = 11) as G3 preoperatively, compared to 66.7% (*n* = 28) as G1 + G2 and 33.3% (*n* = 14) as G3 postoperatively.

**Figure 2:**
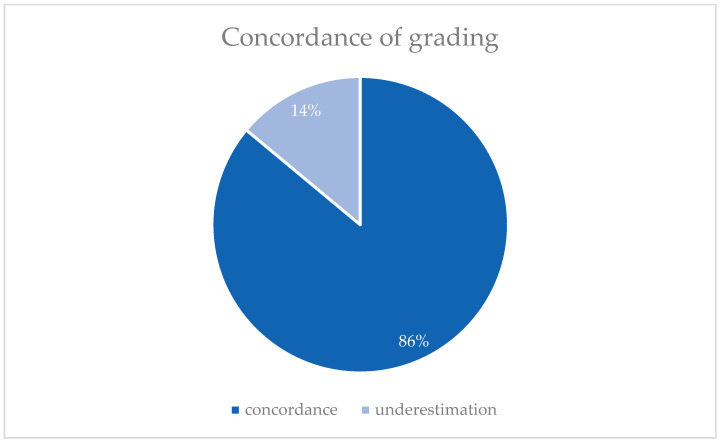
Comparison of pre- and postoperative grading (G) in three category system (*n* = 42).

Comparison of the preoperative and postoperative grading (Figure 2) showed no cases of downgrading, while 14.3% of patients (*n* = 6) experienced postoperative upgrading. A total of 85.7% of patients (*n* = 36) maintained the same grading stage, while 92.9% (*n* = 39) did so in the binary grading system.

**Figure 3:**
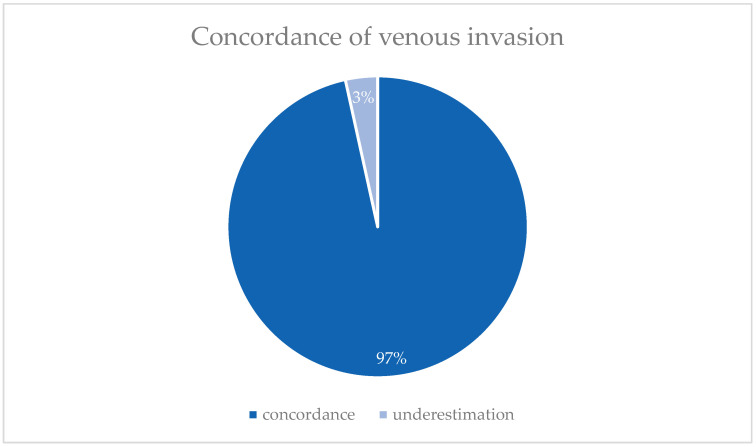
Comparison of pre- and postoperative venous invasion (V) (*n* = 28).

Preoperative venous invasion (V-stage) could not be determined for 14 patients. Among the remaining 28 patients with a known V-stage, all were diagnosed with V0 preoperatively. Postoperatively, the majority (95.2%, *n* = 40) remained at V0, while 4.8% of patients (*n* = 2) were classified as V1. Only one patient (3.6%) showed a discrepancy with a higher postoperative V-stage. For the remaining patients with a known pre- and postoperative V-stage, no discrepancies were observed, as shown in Figure 3.

**Figure 4:**
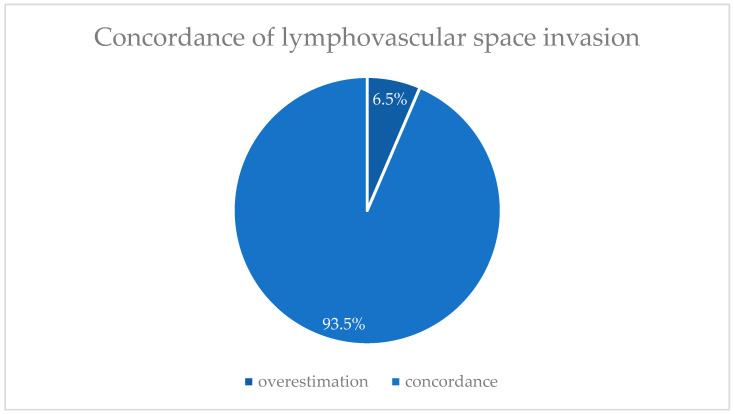
Comparison of pre- and postoperative lymphovascular space invasion (LVSI) (*n* = 30).

Among the 30 patients (71.4%) with a known preoperative LVSI stage, the majority (80.0%, *n* = 24) was classified as L0 and 20.0% of patients (*n* = 6) as L1. Postoperatively, 81.0% of patients (*n* = 34) in the study cohort were classified as L0, while 19.0% (*n* = 8) were classified as L1. A decrease in the LVSI stage was observed in 6.7% of patients (*n* = 2), while no discrepancies were found in 93.3% (*n* = 28), as illustrated in Figure 4.

### 3.3. Correlation of Histopathological Findings, Demographic Data, and Preclinical Risk Factors

To determine whether preclinical risk factors, such as age at first diagnosis, BMI, smoking status, HPV status, and conization, contributed to discrepancies in histological parameters, Fisher’s exact test was performed. No significant differences in the distribution of categories for any variable were detected (Table 3).

Most patients with postoperative upgrading according to the conventional grading model were between 40 and 59 years old (83.3%, *n* = 5). Additionally, 60% of these patients showed obesity (*n* = 3). Although most patients had undergone pre-therapeutic conization, postoperative upgrading was more frequent among those who had not (60%, *n* = 3). A similar trend was observed regarding smoking status, where half of the patients with postoperative upgrading (50%, *n* = 2) were smokers, despite the overall higher proportion of non-smokers. Comparable patterns were observed using the binary grading model: all with postoperative upgrading were aged 40 to 59 years, and most had not undergone pre-therapeutic conization (66.7%, *n* = 2).

### 3.4. Recurrence-Free Survival

The median observation time was 22 months. Throughout the observation period, no patient experienced death; however, 11.9% of patients (*n* = 5) of the study cohort were diagnosed with a recurrence.

**Figure 5:**
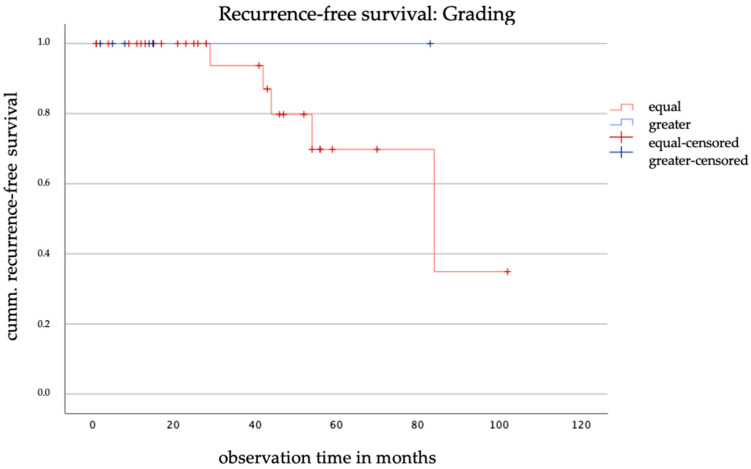
Kaplan–Meier curve of recurrence-free survival based on the discrepancy in grading.

The influence of discrepancies in pre- and postoperative histologic risk factors on recurrence-free survival was analyzed using Kaplan–Meier analysis for both the conventional and binary grading models. Analysis based on the conventional grading model revealed that all recurrences occurred in patients without discrepancies, while no recurrences were observed in those with postoperative upgrading (see Figure 5). The log-rank test yielded a chi-square value of 0.342 and a *p*-value of 0.559, indicating no statistically significant difference in recurrence-free survival between the two groups.

The Kaplan–Meier analysis of the binary grading model showed similar results. According to this classification, all recurrences occurred exclusively in the group of patients without discrepancies, too. Due to the small number of cases and high censoring, neither a chi-square nor a *p*-value could be calculated.

**Figure 6:**
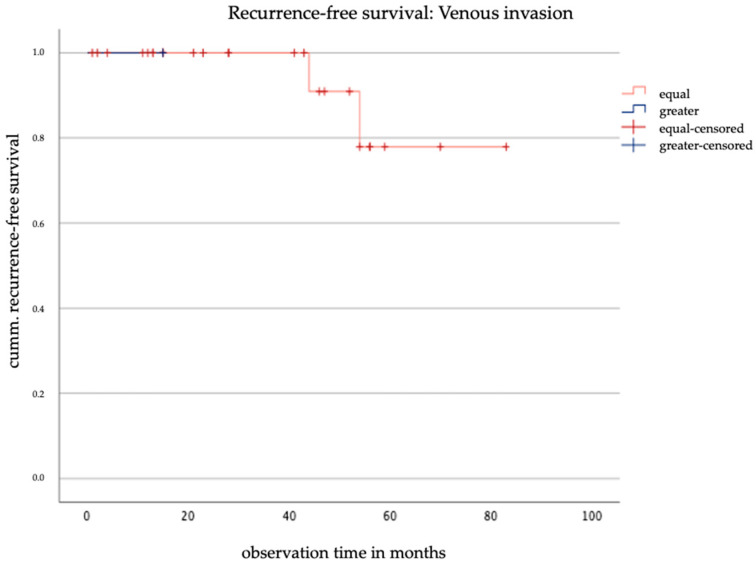
Kaplan–Meier curve of recurrence-free survival based on the discrepancy in venous invasion.

Regarding the influence of discrepancies of venous invasion on recurrence-free survival, the Kaplan–Meier curve indicates that all recurrences occurred in the group without discrepancies, see Figure 6. The small sample size and high censoring rate prevented the calculation of a chi-square value, and a *p*-value could not be calculated.

**Figure 7:**
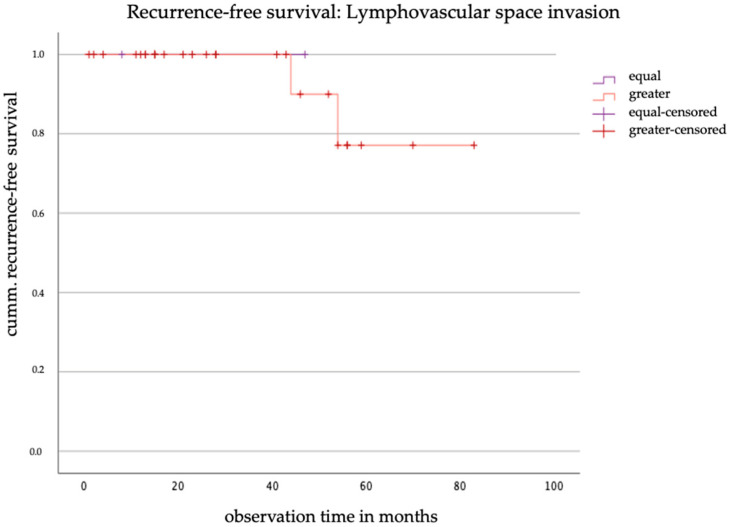
Kaplan–Meier curve of recurrence-free survival based on the discrepancy in lymphovascular space invasion.

In contrast to the analyses of grading and venous invasion, the discrepancy analysis of lymphatic vessel infiltration shows no postoperative upstaging and only a postoperative downstaging of the L-stage. All recorded recurrences (*n* = 2) occurred in the group without discrepancies, see Figure 7. The log-rank test yielded a chi-square value of 0.1 and a *p*-value of 0.752. These results suggest that there is no statistically significant difference in recurrence-free survival between the two groups.

## 4. Discussion

Cervical cancer treatment relies on accurate preoperative staging, but discrepancies between pre- and postoperative assessments can affect outcomes. This study examines the predictive value of preoperative diagnostics, focusing on grading, lymphovascular invasion, and venous invasion, and explores how staging inconsistencies impact survival. Based on data from 42 patients at the University Hospital Cologne, discrepancies between pre- and postoperative findings, their impact on therapeutic decisions, and their effect on recurrence-free and overall survival were investigated.

The median age of onset in the studied cohort was 45 years, which is considerably lower than the median age of onset for cervical cancer in Germany reported to be over 53 years [15]. This lower age may be due to the exclusion of advanced cervical cancers from the cohort, suggesting that women diagnosed with invasive cervical cancer at an early stage tend to be younger than those diagnosed at a more advanced stage.

The median BMI of the cohort was 25.1 kg/m^2^, categorizing the group as overweight and at the borderline of normal weight (cut-off of 25 kg/m^2^). In total, 36.1% of patients were classified as obese (*n* = 13). This reflects trends reported in a comprehensive meta-analysis from 2015, which found that obesity is weakly associated with an increased risk of developing cervical cancer [16].

With the exception of one case, all patients were infected with HPV. This aligns with the findings of Walboomers et al., who identified HPV infection as the most important risk factor and a necessary condition for the development of cervical cancer [17].

The prevalence of nicotine abuse in the present study was 25.7% (*n* = 9), only slightly above the average prevalence in the general German population. Although there are no definitive data on the smoking prevalence specifically among cervical cancer patients in Germany, smoking is known to increase the risk of cervical cancer by approximately 60% [18,19,20].

Several studies have investigated the impact of histopathological risk factors on survival in cervical cancer [21,22,23]. Discrepancies between preoperative and postoperative histological findings have also been studied, particularly in terms of differences between clinical and pathological tumor staging [10,11,12,13,24]. A study conducted by Chen et al. demonstrated that inconsistencies between clinical and pathological tumor stages in early-stage cervical cancer carry prognostic significance. Patients whose tumors were clinically underestimated had a higher recurrence rate and worse overall survival compared to those with accurate or overestimated staging [10]. Similar results were found by Toure et al., who examined discrepancies between pre- and postoperative tumor stages using the FIGO classification [13]. They showed that poor agreement lead to frequent clinical underestimation and incorrect treatment decisions and resulted in faster postoperative progression and worse prognosis.

Additionally, several studies have also reported differing results regarding the prognostic value of tumor grade [4]. Currently, conventional grading does not include nuclear morphology, which may lead to insufficient prognostic utility. Horn et al. proposed a binary grading model (G1 + G2 vs. G3) demonstrating higher prognostic significance than traditional grading. To improve risk stratification, there is ongoing discussion about the implantation of a binary grading model in squamous cell carcinoma, classifying tumors as low- or high-grade [25].

The effect of grading discrepancies on recurrence-free survival in cervical cancer has not yet been studied. However, such discrepancies have been investigated in other gynecologic malignancies [5,6,26]. Helpman et al. found that, in endometrial cancer, postoperative upgrading occurred in 36% of cases, while 23% experienced downgrading [5]. These variations were attributed to the limitations of preoperative biopsies, which often fail to reflect the complete tumor histology and may underestimate nodal spread or recurrence risk. Lago et al. reported similar findings in endometrioid endometrial carcinomas, with a grading accuracy of 75% for G1, 73% for G2, and 90% for G3. Upgrading was found in 16% and downgrading in 12% of cases, highlighting the role of intratumoral heterogeneity and limited biopsy material as contributing factors [26].

A systematic review of breast cancer found a pooled agreement rate of only 71.7% core needle biopsy and postoperative histology. Preoperative underestimation occurred more frequently than overestimation, likely due to inadequate biopsy material. The authors also noted that technical factors, such as cold ischemia time, fixation methods, and temperature, may contribute to grading discrepancies [6].

In the present study, no preoperative overestimation of tumor grading was observed. However, 14.3% of patients (*n* = 6) showed preoperative underestimation using the conventional grading model and 7.1% (*n* = 3) using the binary grading model, consistent with prior research. No significant correlation was found between grading discrepancies and preoperative histological confirmation via conization. Interestingly, postoperative upgrading was more frequent in women without preoperative conization, suggesting that standardized conization prior to definitive treatment may reduce grading inconsistencies. Future research should further explore how different biopsy techniques influence grading accuracy in cervical cancer.

Only one patient showed a discrepancy in venous invasion, with a higher postoperative V-stage. This patient was over 60 years old, obese, HPV-16 positive, a non-smoker, and did not undergo pre-therapeutic conization. Due to the small sample size, meaningful conclusions concerning lymphatic vessel invasion are difficult to draw, but it is worth noting that both patients with a lower postoperative LVSI stage had undergone pre-therapeutic conization, which may indicate a potential association worth exploring in larger cohorts.

Among patients with grading discrepancies (both grading models), 50% were smokers, although the majority of the total cohort were non-smokers. While this finding was not statistically significant (*p* = 0.268), it points to a possible link that should be further explored in larger studies.

No association was found between grading discrepancies and recurrence-free survival, consistent with findings in endometrial cancer, where grading inconsistencies did not significantly affect overall survival [5]. All observed recurrences occurred in the group without grading discrepancies, suggesting that additional factors may play a role in prognosis. The small sample size of this study may also have limited the ability to detect significant differences.

The prognostic relevance of lymphovascular space invasion (LVSI) and venous invasion in cervical cancer remains controversial. In this cohort, no significant impact of LVSI discrepancies on recurrence-free survival was observed, and only one patient exhibited a discrepancy in venous invasion. Given the rarity of venous invasion, further studies with larger sample sizes are needed to better evaluate the prognostic significance of these factors.

The findings must be considered within the context of certain limitations. Singh et al. emphasized the challenges of predicting tumor behavior based on histopathology, given the variability in definitions and criteria [22,23]. Similarly, van Bommel et al. highlighted the interdependent nature of histopathological factors, suggesting that their prognostic value is only meaningful when evaluated in combination [22]. Both studies conclude that the combination of multiple factors shows a worse prognosis and that only such a combination allows for an accurate assessment of the tumor. This is reflected in current clinical guidelines, which recommend basing treatment decisions on the presence of at least two risk factors.

Additionally, the limited number of events in the present study cohort poses a further limitation, as small sample sizes may result in false-negative findings and obscure relevant associations.

The observed discrepancies between preoperative and postoperative grading, particularly the tendency for underestimation, highlight the need for caution when making treatment decisions based solely on preoperative biopsies. In cases where the clinical suspicion is high but the biopsy indicates a low-grade tumor (G1–G2), a repeat biopsy or the use of advanced imaging techniques such as MRI may be warranted. These approaches could help reduce the risk of under-treatment by providing a more accurate assessment of tumor aggressiveness and guiding appropriate therapeutic strategies.

The relatively small cohort size limits the statistical power of our analyses and may hinder the detection of clinically relevant differences. As such, the findings should be interpreted with caution, and validation in larger, prospective studies is necessary to strengthen the evidence base.

## 5. Conclusions

In summary, our study revealed that discrepancies in histological risk factors (G, V, LVSI) did not significantly affect recurrence-free survival. The results are likely influenced by the challenge of isolating individual histopathological factors, as many of them are interrelated. The combination of multiple factors may provide a more accurate tumor assessment, as supported by current guidelines. To substantiate our results and improve their applicability to broader clinical settings, further research involving larger patient populations and extended follow-up is essential.

## Figures and Tables

**Table 1:** Patients’ demographic data, preclinical risk factors, and tumor data: quantitative data are presented as median [Q25–Q75] and qualitative data as frequency (counts).

	Total (*n* = 42)
Age at first diagnosis (years)	

	45 [36–54]
BMI (kg/m^2^) unknown	25.1 [20.7–26.4] 6

Smoking yes no unknown	
	9 (25.7%)
	26 (74.3%)
	7
HPV negative HPV 16-positive HPV 18-positive other high-risk HPV other low-risk HPV unknown	1 (3.6%) 18 (64.3%) 2 (7.1%) 6 (21.4%) 1 (3.6%)
	14
Cone biopsy yes no unknown	25 (62.5%) 15 (37.5%) 2
Clinical FIGO stage FIGO I FIGO II unknown	23 (85.2%) 4 (14.8%) 15
Postoperative T stage pT1 pT2 unknown	40 (95.2%) 2 (4.8%) 0
Postoperative N stage pN0 pN1 unknown	36 (97.3%) 1 (2.7%) 5
Clinical M stage cM0 unknown	32 (100%) 10

**Table 2:** Pre- and postoperative findings in grading, venous invasion, and lymphovascular space invasion.

		Preoperative	Postoperative
Grading	G1	6 (14.3%)	3 (7.1%)
	G2	25 (59.5%)	25 (59.5%)
	G3	11 (26.2%)	14 (33.3%)
Venous invasion	V0	28 (100%)	40 (95.2%)
	V1	0	2 (4.8%)
	unknown	14	0
Lymphovascular space invasion	L0	24 (80.0%)	34 (81.0%)
	L1	6 (20.0%)	8 (19.0%)
	unknown	12	0

**Table 3:** Correlation between the discrepancy of pre- and postoperative grading (G), venous invasion (V), and lymphovascular space invasion (LVSI) and preclinical risk factors, resulting in postoperative equal (concordance), higher (underestimation) or lower (overestimation) histopathological results.

	G (*n* = 42)			V (*n* = 28)			LVSI (*n* = 30)		
	Equal	Higher	*p* Value	Equal	Higher	*p* Value	Lower	Equal	*p* Value
Age (years)									
<40	17 (47.2%)	-	0.075	13 (48.1%)	-	0.107	-	14 (50.0%)	0.372
40–59	14 (38.9%)	5 (83.3%)		12 (44.4%)	-		2 (100%)	11 (39.3%)	
>60	5 (13.9%)	1 (16.7%)		2 (7.4%)	1 (100%)		-	3 (10.7%)	
BMI									
underweight	1 (3.2%)	-	0.490	-	-	0.208	-	-	0.697
normal	20 (64.5%)	2 (40%)		13 (56.5%)	-		1 (50.0%)	13 (56.5%)	
overweight	10 (32,3%)	3 (60%)		10 (43.5%)	1 (100%)		1 (50.0%)	10 (43.4%)	
unknown	5	1		4	-		-	5	
HPV									
negative	1 (4.0%)	-	1	-	-	1	-	-	1
HPV16	16 (64.0%)	2 (66.7%)		14 (70%)	1 (100%)		2 (100%)	14 (70.0%)	
HPV18	2 (8.0%)	-		2 (10%)	-		-	2 (10.0%)	
other HPV	6 (24.0%)	1 (33.3%)		4 (20%)	-		-	4 (20.0%)	
unknown	11	3		7	-		-	8	
Smoking									
yes	7 (22.6%)	2 (50%)	0.268	4 (18.2%)	-	1	1 (50.0%)	4 (18.2%)	0.380
no	24 (77.4%)	2 (50%)		18 (81.8%)	1 (100%)		1 (50.0%)	18 (81.8%)	
unknown	5	2		5	-		-	6	
Conization									
yes	23 (65.7%)	2 (40%)	0.345	21 (77.8%)	-	0.250	2 (100%)	21 (75.0%)	1
no	12 (34.3%)	3 (60%)		6 (22.2%)	1 (100%)		-	7 (25.0%)	
unknown	1	1		-	-		-	-	

## Data Availability

The original data presented in this study are available from the corresponding author upon reasonable request.

## Abbreviations

The following abbreviations are used in this manuscript:

G	Grading
L	Lymphovascular space invasion
N	Regional lymph node involvement
M	Metastases
V	Venous invasion
